# Dietary Supplementation of *Caulerpa racemosa* Ameliorates Cardiometabolic Syndrome via Regulation of PRMT-1/DDAH/ADMA Pathway and Gut Microbiome in Mice

**DOI:** 10.3390/nu15040909

**Published:** 2023-02-11

**Authors:** Fahrul Nurkolis, Nurpudji Astuti Taslim, Dionysius Subali, Rudy Kurniawan, Hardinsyah Hardinsyah, William Ben Gunawan, Rio Jati Kusuma, Vincentius Mario Yusuf, Adriyan Pramono, Sojin Kang, Nelly Mayulu, Andi Yasmin Syauki, Trina Ekawati Tallei, Apollinaire Tsopmo, Bonglee Kim

**Affiliations:** 1 Department of Biological Sciences, Faculty of Sciences and Technology, State Islamic University of Sunan Kalijaga (UIN Sunan Kalijaga), Yogyakarta 55281, Indonesia; 2 Division of Clinical Nutrition, Department of Nutrition, Faculty of Medicine, Hasanuddin University, Makassar 90245, Indonesia; 3 Department of Biotechnology, Faculty of Biotechnology, Atma Jaya Catholic University of Indonesia, Jakarta 12930, Indonesia; 4 Department of Internal Medicine, Faculty of Medicine, University of Indonesia—Cipto Mangunkusumo Hospital, Jakarta 10430, Indonesia; 5 Division of Applied Nutrition, Department of Community Nutrition, Faculty of Human Ecology, IPB University, Bogor 16680, Indonesia; 6 Department of Nutrition Science, Faculty of Medicine, Diponegoro University, Semarang 50275, Indonesia; 7 Department of Nutrition and Health, Faculty of Medicine, Public Health, and Nursing, Gadjah Mada University, Yogyakarta 55223, Indonesia; 8 Center for Herbal Medicine, Faculty of Medicine, Public Health, and Nursing, Universitas Gadjah Mada, Yogyakarta 55223, Indonesia; 9 Medical Study Programme, Faculty of Medicine, Brawijaya University, Malang 65145, Indonesia; 10 Department of Pathology, College of Korean Medicine, Kyung Hee University, Kyungheedae-ro 26, Dongdaemun-gu, Seoul 05254, Republic of Korea; 11 Department of Nutrition, Faculty of Medicine, Sam Ratulangi University, Manado 95115, Indonesia; 12 Department of Biology, Faculty of Mathematics and Natural Sciences, Universitas Sam Ratulangi, Manado 95115, Indonesia; 13 Food Science and Nutrition Program, Department of Chemistry, Carleton University, 1125 Colonel by Drive, Ottawa, ON K1S 5B6, Canada; 14 Institute of Biochemistry, Carleton University, Ottawa, ON K1S 5B6, Canada

**Keywords:** cardiometabolic syndrome, *Caulerpa racemosa*, PRMT-1/DDAH/ADMA pathway, gut microbiota, cardioprotective, sea grapes, nutraceuticals, seaweed, green algae

## Abstract

This study evaluated the effects of an aqueous extract of *Caulerpa racemosa* (AEC) on cardiometabolic syndrome markers, and the modulation of the gut microbiome in mice administered a cholesterol- and fat-enriched diet (CFED). Four groups of mice received different treatments: normal diet, CFED, and CFED added with AEC extract at 65 and 130 mg/kg body weight (BW). The effective concentration (EC_50_) values of AEC for 2,2-diphenyl-1-picrylhydrazyl (DPPH), 2,2′-azino-bis(3-ethylbenzothiazoline-6-sulfonic acid) (ABTS), and lipase inhibition were lower than those of the controls in vitro. In the mice model, the administration of high-dose AEC showed improved lipid and blood glucose profiles and a reduction in endothelial dysfunction markers (PRMT-1 and ADMA). Furthermore, a correlation between specific gut microbiomes and biomarkers associated with cardiometabolic diseases was also observed. In vitro studies highlighted the antioxidant properties of AEC, while in vivo data demonstrated that AEC plays a role in the management of cardiometabolic syndrome via regulation of oxidative stress, inflammation, endothelial function (PRMT-1/DDAH/ADMA pathway), and gut microbiota.

## 1. Introduction

Cardiometabolic syndrome is a group of metabolic disorders that increase the risk of developing cardiovascular diseases. Metabolic dysfunctions associated with the syndrome include insulin resistance, glucose intolerance, atherogenic dyslipidemia, hypertension, and intra-abdominal adiposity. The prevalence of cardiometabolic syndrome ranges from 7% to 45% [1]. The discovery of a novel cardiometabolic syndrome therapy is challenging due to a multitude of etiopathogenesis factors, including the diabetes triumvirate (insulin resistance in muscles, liver, and pancreatic beta cell failure), omnibus octet, egregious eleven, dirty dozen, treacherous thirteen, formative fifteen, microbiota dysbiosis, and intestinal barrier dysfunction [2,3,4]. The complexity of the syndrome makes the available therapy costly and also limits its availability [5]. Hence, countries with higher per capita incomes tend to get therapy more easily [6]. Therapeutic innovations based on bioresources can offer an alternative approach that is easily accessible and relatively inexpensive to help control metabolic syndrome. As a tropical and archipelagic country, Indonesia has a wealth of natural products that can be used as health agents, such as nutraceuticals. Some of these products are algae and seaweed [7].

Indonesia has more than 30,000 species of plants and marine resources [7], of which many have been only partially investigated or not studied at all [7]. Our group has directed its attention to seaweed as a potential source of functional ingredients with health benefits. Unlike terrestrial plants, marine algae have not been widely used as an alternative medicine or adjuvant to medicines [7]. *Caulerpa racemosa* (green seaweed, known as “sea grapes”) is widely distributed in tropical regions, notably in Indo-Pacific Asia, and is a member of the *Caulerpaceae* family [8]. This species contains peptides, fibers (polysaccharides), polyphenols, and flavonoids, which are known to possess diverse biological activities [9,10]. Consumption of food rich in polyphenols and other antioxidant molecules may attenuate cardiometabolic syndrome [11]. Previous studies on *C. racemosa* have reported anti-obesity and antiaging effects through the modulation of blood glucose and lipid profiles [12,13,14]. However, to date, no studies have successfully reported the effects of dietary supplementation of *Caulerpa racemosa* on the markers of cardiometabolic syndrome. Therefore, the aim of this study was to determine the effect of *C. racemosa* on balancing the markers of the cardiometabolic syndrome in a mice model. The markers investigated are related to oxidative stress, inflammation, endothelial function, digestion of sugars and lipids, and modulation of the gut microbiome.

## 2. Materials and Methods

### 2.1. Collection and Preparation of Caulerpa Racemosa

Green sea grapes (*Caulerpa racemosa*) were obtained from the *C. racemosa* Aquaculture Pond Waters in Jepara Regency, Central Java Province, Indonesia (6°35′12.5″ S latitude; 110°38′36.0″ E longitude). Once obtained, *C. racemosa* samples were washed immediately to remove dirt. The authentication and identification of the botanical species were carried out in the laboratory of Biological Sciences, Faculty of Science and Technology, State Islamic University of Sunan Kalijaga (UIN Sunan Kalijaga), Yogyakarta, Indonesia, and complied with the National Center for Biotechnology Information (NCBI) Taxonomy ID 76,317 (Eukaryota/Viridiplantae/Chlorophyta/Ulvophyceae/Bryopsidales/Caulerpaceae/Caulerpa). The whole body of the collected specimen was thoroughly rinsed with distilled water, dried in a hybrid hot water Goodle dryer, and lyophilized (Lyovapor™ L-200 by BÜCHI Labortechnik AG, Flawil, Switzerland). The obtained dehydrated coarse powder was then ground, as reported in previous studies [12,13,15]. The fine powder was packed in aluminum foil and deep-frozen at −20 °C before use for subsequent experiments.

### 2.2. Preparation of the Aqueous Extract of Caulerpa Racemosa (AEC)

The procedure for aqueous extraction was adapted from previous studies [16,17]. *C. racemosa* fine powder samples were mixed with distilled water at a solid-liquid ratio of 1:19 and sonicated (60 min, 40 °C) using an ultrasound sonicator (400 W, Branson 2510 model; Danbury, CT, USA). After filtration, the AEC powder was stored airtight in aluminum foil at −20 °C until use for in vitro and in vivo tests. The concentration of caulerpin, the major bioactive secondary metabolite (e.g., antioxidant and anti-inflammatory effects) of *Caulerpa sp.,* was quantified by high-performance liquid chromatography-mass spectrometry (HPLC-MS), as shown in Supplementary Table S1 [12].

### 2.3. In Vitro Studies

#### 2.3.1. Antioxidant Activity by ABTS and DPPH Radical Scavenging Activity Assays (ABTS and DPPH Inhibition, %)

The scavenging of 2,2′-azino-bis(3-ethylbenzothiazoline-6-sulfonic acid) or its diammonium salt radical cation (ABTS+; Sigma-Aldrich, Saint Louis, MO, USA) was determined based on a published procedure [18,19]. Potassium persulfate (2.4 mM) and 7 mM ABTS (7 mM) were mixed at a ratio of 1:1, protected from light with aluminum foil, and allowed to react for 14 h at 22 °C. The mixture was further diluted (e.g., 1 mL of the stock solution plus 60 mL of ethanol) to obtain a working solution with an absorbance of 0.706 at 734 nm. A fresh working solution was prepared for each test. The AEC extracts (50, 100, 150, 200, and 250 μg/mL) were diluted with ABTS working solution (1 mL), and the absorbance was measured at 734 nm after 7 min. Trolox, a known antioxidant molecule, was used as a positive control.

The antioxidant activity in the 2,2-diphenyl-1-picrylhydrazyl radical-scavenging activity (DPPH) test was assayed according to [12,18,19]. In the testing vial, a concentration of 50, 100, 150, 200, and 250 μg/mL of AEC was added to the DPPH reagent (3 mL). The DPPH-AEC mixture was then cooled at room temperature for 30 min. A change in the concentration of DPPH was observed based on 517 nm absorbance. Glutathione (GSH; Sigma-Aldrich, 354102) was used as a positive control.

To ensure the validity of the data results (ABTS and DPPH tests), each sample was analyzed in triplicate (*n* = 3).

Inhibition of DPPH and ABTS was expressed as a percentage and determined according to the formula below:

(1) $$Inhibition\;Activity\;\left(\%\right)=\frac{A0-A1}{A0}\times 100\%\;$$

*A*0 = absorbance of the blank; *A*1 = absorbance of the standard or sample.

The half-maximal effective concentration ratio (EC_50_) was used to express the radical-scavenging capability of AEC and Trolox for ABTS and AEC and glutathione for the DPPH assay. EC_50_ was defined as the concentration of a sample that caused a 50% decrease in the initial radical concentration.

#### 2.3.2. α-Glucosidase Inhibition Assay

The inhibitory activity was evaluated according to the literature [19]. The enzyme (1 mg, 76 UI) was mixed with 50 mL of phosphate buffer (pH 6.9) to obtain a concentration of 1.52 UI/mL. In the reaction tube, 0.35 mL of sucrose (65 mM), maltose solution (65 mM), and AEC extract (0.1 mL of 50, 100, 150, 200, and 250 μg/mL) were added, one at a time. After homogenization, α-glucosidase solution (1.52 UI/mL, 0.2 mL) was added to each tube, which was then maintained at 37 °C for 20 min. The enzyme was heat-inactivated in a water bath for 2 min at 100 °C. Acarbose served as the positive control. To develop the color, 0.2 mL of testing solution and color reagent (3 mL) were reacted. Next, the system was heated (37 °C) for 5 min, and the absorbance of the solution was examined at 505 nm. The amount of glucose released during the reaction served as a sign of inhibitory activity.

#### 2.3.3. α-Amylase Inhibition Assay (%)

The **α**-amylase inhibition activity of the AEC extract was measured based on a published method [12]. Diluted AEC at five different concentrations (50, 100, 150, 200, and 250 μg/mL) was incubated for 10 min at 25 °C with sodium phosphate buffer (0.02 M, pH 6.9), 0.006 M NaCl, and 0.5 mg/mL of porcine pancreatic amylase. Then, 500 µL of a 1% starch solution in assay buffer was added to each mixture. After 10 min of incubation at 25 °C, 3,5-dinitrosalicylic acid was added to complete the process and incubated in a water bath at 100 °C for 5 min. The test tube was then allowed to cool down to 22 °C. To obtain the values in the permissible range for recording the absorbance at 540 nm, a dilution with distilled water (10 mL) was performed. Acarbose was used as the positive control.

#### 2.3.4. Lipase Inhibition Assay (%)

Crude pig pancreatic lipase (PPL, 1 mg/mL) was first dissolved in phosphate buffer (50 mM, pH 7) before centrifugation at 12,000× *g* to remove insoluble components. Preparing an enzyme stock (0.1 mg/mL) required a 10-fold dilution of the supernatant with buffer. The lipase inhibition potential was assessed based on prior research [20]. A transparent 96-well microplate containing 100 µL of AEC was combined with 20 µL of p-nitrophenyl butyrate (pNPB,10 mM in buffer) and incubated for 10 min at 37 °C. The outcome was compared to the reference drug orlistat, a well-known PPL inhibitor. Measurements were taken at 405 nm using a microplate reader. The unit of activity was calculated using the yield from the reaction rate of 1 mol of p-nitrophenol/min at 37 °C. To measure the lipase inhibition activity, PPL activity was reduced in the test mixture by a specific amount. To ensure the validity of the study results, each sample was verified in triplicate. The inhibitory data were obtained using the equation below.

(2) $$Inhibition\;of\;Lipase\;Activity\;\left(\%\right)=100-\frac{B-Bc}{A-Ac}\times 100\%\;$$

*A* = activity without inhibitor; *Ac* = negative control without inhibitor; *B* = activity with inhibitor; *Bc* = negative control with inhibitor.

### 2.4. In Vivo Study Design

#### 2.4.1. Animal Handling and Ethical Approval

Forty male albino Swiss mice (*Mus musculus*), each weighing 21.53 ± 1.92 g (3–5 weeks old), were provided by Animal Model Farm Yogyakarta, Indonesia, and then shipped to the research site. Mice were housed in cages and kept in a climate-controlled environment (27 °C, 50–60% relative humidity) with a balanced light-dark cycle. All mice were acclimated in the lab for 10 days before the experiment. During the research, mice had unrestricted access to conventional animal feed or pellets from PT Citra Ina Feedmill as well as drinking water. The mice were randomly divided into four treatment groups after the 10-day acclimatization period. The animal research protocol used mentions the Declaration of Helsinki and the Council of International Organizations of Medical Sciences (CIOMS). In addition, all procedures involving animals adhered to the Guidelines for Reporting In Vivo Experiments (ARRIVE) and obtained approval from the ethics board of the International Register of Preclinical Trial Animal Studies Protocols (preclinicaltrials.eu) with registration number PCTE0000329.

#### 2.4.2. Study Design of Treatments

Throughout the study, skilled veterinarians looked out for any signs of animal welfare problems, such as lack of food, ruffling, lethargy, indifference, hiding, or curling up. Moreover, mice got weekly examinations for specific health and weight loss markers. Forty mice were randomly divided into four treatment groups. Based on Federer’s equation, a minimum sample of six is required for an animal experiment consisting of four groups. Ten mice were used in this study as backup and for further sample harvest. Group A was given a normal diet and water ad libitum. Group B was given a cholesterol- and fat-enriched (CFED) diet with ad libitum water. Groups C and D were given a CFED diet and water ad libitum with daily supplementation of 65 and 130 mg/kg of body-weight (BW) AEC, respectively. An expert administered the AEC dosages orally. The daily consumption of animal feed and drinking water was tracked during the entire experiment; therefore, there was no difference between the control and experimental groups.

#### 2.4.3. Feed or Pellet Composition and CFED Production

Normal pellets contained 12% moisture, 20% protein, 4% fat, 14% calcium, 1% fiber, 0.7% phosphorus, 11.5% total ash, 0.3% vitamin C, and 0.1% vitamin E. These pellets were obtained from Rat Bio^®^ (Citra Ina Feedmill Ltd, Jakarta, Indonesia). Pellets were kept out of direct sunlight and were kept cool and dry according to the manufacturer’s recommendations.

The CFED diet was prepared following earlier research [13,20]. Cholic acid (1%), cholesterol powder (2%), animal fat (20%), and maize oil (2%) were added to dry, normal pellets. After the ingredients had been homogenized, 1 L of distilled water was added to the mixer, where the pellets were subsequently shaped into smaller pieces. To reduce oxidation, the pellets were first dried under sterile conditions at ambient temperature before storage at 4 °C. Specifically, 43.6% of CFED is composed of carbohydrates, while 12.4% is protein, 4.7% is fiber, 3.2% is fat, 2% is cholesterol, 1% is cholic acid, 20% is animal fat, 4% is total ash, 2% is maize oil, and the remainder is moisture.

#### 2.4.4. Biomedical Analysis of Collected Blood Samples

Blood was taken six weeks after the mice underwent interventional feeding. The night before blood was obtained, the animals were fasted and administered ketamine as an anesthetic. Blood was drawn from the venous sinus, and after being placed in a sterile, dry tube devoid of any anticoagulant, it was allowed to coagulate at room temperature. The serum was then obtained after 20 min of centrifugation (3000 rpm). Low-density lipoprotein (LDL), triglycerides (TG), high-density lipoprotein (HDL), total cholesterol (TC), and blood glucose (BG) biomedical analyses were carried out using the COBAS Integra^®^ 400 Plus Analyzer (Roche Diagnostics, Basel, Switzerland). Blood was also taken from the heart tissue to evaluate other biomarkers such as superoxide dismutase (SOD) enzyme activities using the SOD Assay Kit from Sigma-Aldrich, serum lipase levels using the Mouse Pancreatic Lipase ELISA Kit (Merck KGaA, Darmstadt, Germany), and serum amylase levels using the Mouse Pancreatic Amylase ELISA Kit. Inflammatory biomarkers, namely PGC-1α (peroxisome proliferator-activated receptor-gamma coactivator-1 alpha), TNF-α (tumor necrosis factor-alpha), and IL-10 (interleukin 10), were quantified using a PGC-1α Mouse ELISA Kit (Sunlong Biotech Co., Ltd.; Zhejiang, China), a Mouse Tumor Necrosis Factor-α (TNF-α) Kit (Sunlong Biotech Co., Ltd.; Zhejiang, China), and an IL-10 ELISA Kit (Abcam), respectively. For the cardiometabolic biomarkers, both PRMT-1 (protein arginine N-methyltransferase 1) and DDAH-II (dimethylarginine dimethylaminohydrolase 2) were quantified using an LS-F65223 ELISA Kit and an LS-F14238 ELISA Kit, both from LSBio (Seattle, WA, USA). ADMA (asymmetric dimethylarginine) was evaluated directly using an ALX-850-327-KI01 ADMA Direct Mouse ELISA Kit from Enzo Life Sciences, Inc. (New York, NY, USA). Digital scales were used to determine the body weights of the mice.

### 2.5. Gut Microbiota Sequencing and Analysis of the 16S rRNA Gene in Mice Feces

Microbiological genomes of intestinal bacteria were extracted from excrements using OMG soil extraction kits from Shanghai Meiji Biopharmaceutical Technology Co., Ltd (Shanghai, China). Polymerase chain reaction (PCR) amplification of the V3–V4 variable region of the 16S rRNA gene was performed using primers 338F (5′-ACTCCTACGGGAGGCAGCAG-3′) and 806R (5′-GGACTACHVGGGTWTCTAAT-3′). Sequencing was carried out using the Illumina Miseq PE300 platform. The raw sequences were controlled with FAST software, cut with FLASH software, and qualified reads were clustered with UPARSE software to generate Operational Taxonomic Units (OTUs) with 97% similarity. Chimera sequences were removed using the search software. Each sequence was classified as a species using ribosomal database project (RDP) classification, and a 70% comparison threshold to the Silva 16S rRNA database (Version 138 by Max Planck Institute for Marine Microbiology and Jacobs University, Bremen, Germany) was set.

### 2.6. Data Analysis and Management

An unpaired *t*-test was used to statistically assess the data from in vitro experiments, including antioxidant inhibition of ABTS and DPPH and lipase, α-amylase, and α-glucosidase inhibitory activities. Experiments were performed in triplicate. Each EC_50_ data set was created using nonlinear regression formulas. In vivo lipid profile (LDL, HDL, TG, TC, and BG), inflammatory biomarkers (IL-10, TNFα, and PGC-1α), enzymatic assays (SOD cardio, serum lipase, and serum amylase), and cardiometabolic biomarkers (PRMT-1, DDAH-II, and ADMA) were analyzed using multivariate ANOVA. To identify significant variations between the starting and ending body weights (g) in each group, paired t-tests or dependent t-tests were used. In addition, one-way ANOVA was conducted to find the differences between each secondary parameter (water intake (mL), food intake (g), the food efficiency ratio (FER), and the beginning, final, and daily weight gain (g/day)) for each group. All data were provided in the form of mean ± standard error of the mean (SEM) with a 95% confidence level. On MacBook computers, GraphPad Prism version 9.4.1 software (Boston, MA, USA) was used for all in vitro and in vivo data analyses.

## 3. Results

### 3.1. In Vitro Activities of the Aqueous Extract of Caulerpa racemosa (AEC)

**Figure 1:**
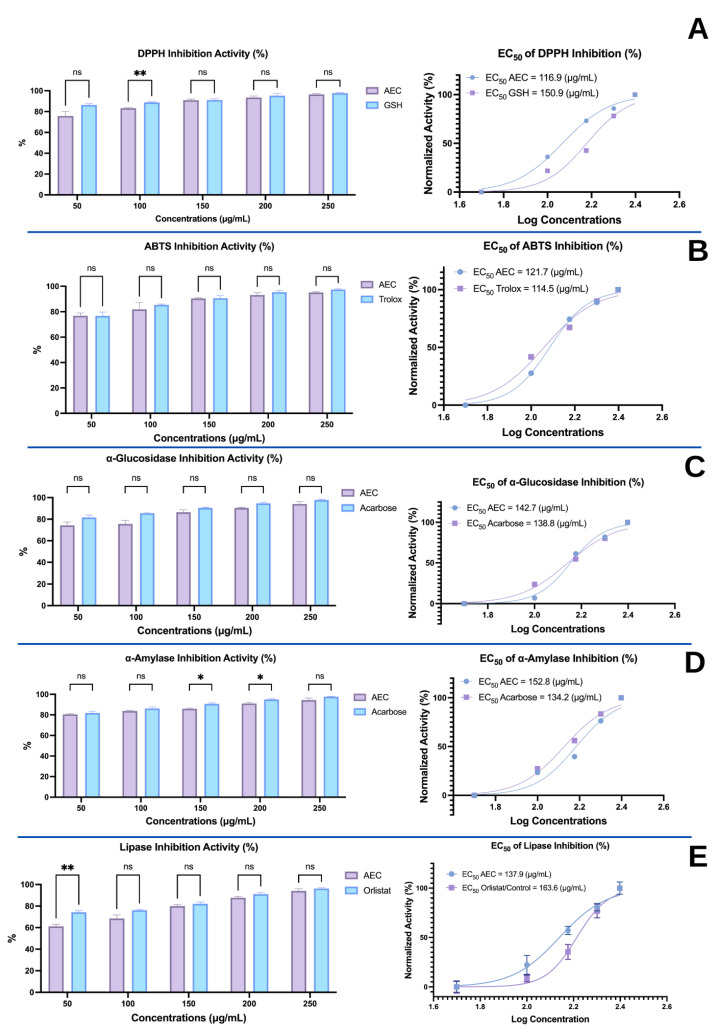
In vitro activities of the aqueous extract of *Caulerpa racemosa* (AEC). (**A**) DPPH radical-scavenging activity. (**B**) ABTS radical-scavenging activity. (**C**) Inhibition of the α-glucosidase enzyme. (**D**) Inhibition of the α-amylase enzyme. (**E**) Inhibition of the lipase activity; ns: *p* = 0.1638 (*p* > 0.05); *: *p* = 0.0125; **: *p* = 0.0007. Note: The EC_50_ was analyzed using the GraphPad Premium statistical analysis package “non-linear regression (log(inhibitor) vs. normalized response—variable slope”. The plot bars showed that at a concentration of 50 μg/mL, all inhibition activity exceeded 50%. This may be caused by the unequal maximum and baseline effects of AEC compared to the controls. For further research, using a lower minimum dose for the controls and a higher maximum amount of AEC may result in a comparable dose response for all compounds (EC_50_).

The in vitro activities of extracts and compounds can provide an indication of their properties in biological systems. As such, the antioxidant activities (DDPH and ABTS) and enzyme-inhibitory properties (α-glucosidase, α-amylase, and lipase) of the AEC extract were obtained prior to in vivo tests. Data reported as half-maximal effective concentration (EC_50_) or half-maximal inhibitory concentration (IC_50_) values are shown in Figure 1.

In the 2,2-diphenyl-1-picrylhydrazyl (DPPH) antioxidant assay, glutathione (GSH, control) had a greater inhibitory effect than AEC at a concentration of 100 μg/mL (*p* < 0.05; Figure 1A). However, at other concentrations, there were no significant differences (*p* > 0.05). DPPH overall suggests that there is no difference in the scavenging power of the AEC extract relative to GSH. Furthermore, the EC_50_ value of AEC (116.9 μg/mL) was lower than that of GSH (150.9 μg/mL), suggesting that AEC is more effective in inhibiting DPPH than GSH (Figure 1A).

Trolox (control) had greater inhibitory activity against ABTS compared to AEC at all concentrations (100–250 μg/mL). Nevertheless, scavenging activities were comparable (*p* > 0.05; Figure 1B). Similar to the DPPH inhibition results, the EC_50_ AEC values are more potent and active together with Trolox, with values of 121.7 μg/mL and 114.5 μg/mL, respectively (Figure 1B).

The abilities of the AEC extract to inhibit α-glucosidase (Figure 1C) and α-amylase (Figure 1D) were both compared to the reference inhibitor acarbose. At all concentrations (50–250 μg/mL), the inhibition of α-glucosidase by AEC was not significantly different compared to acarbose (*p* > 0.05), which indicates that AEC has an activity equivalent to that of the acarbose control (Figure 1C). Regarding α-amylase, AEC and acarbose had equivalent inhibition at 50 μg/mL, 100 μg/mL, and 250 μg/mL (Figure 1D), while acarbose was superior at the other two concentrations. For both enzymes, the EC_50_ of AEC was greater than that of the control acarbose.

The inhibition of pancreatic lipase by AEC did not differ significantly from the positive control (orlistat) at 100 μg/mL to 250 μg/mL (*p* > 0.05). Nevertheless, at the lowest concentration, the positive control had greater activity (Figure 1E). Based on the EC_50_ values, AEC is more potent at inhibiting the lipase enzyme (EC_50_ AEC < EC_50_ orlistat; Figure 1E).

### 3.2. Effects of the Extract of Caulerpa Racemosa (AEC) on the Cardiometabolic Markers in Mice

**Figure 2:**
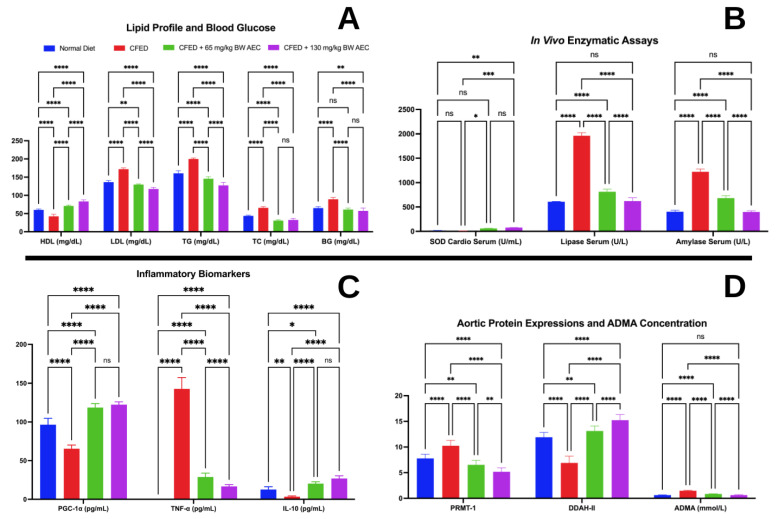
Effect of the aqueous extract of *Caulerpa racemosa* (AEC) on cardiometabolic markers. (**A**) Improvement of blood lipid and glucose profile of CFED mice by AEC. (**B**) Significance of improvements in SOD in cardio serum, lipase, and amylase in serum. (**C**) Improvement of inflammatory markers in CFED mice by dietary AEC. (**D**) Amelioration of cardiometabolic syndrome via regulation of PRMT-1/DDAH/ADMA pathway. ns: *p* = 0.1638 (*p* > 0.05); *: *p* = 0.0125; **: *p* = 0.0067; ***: *p* = 0.0007; ****: *p* < 0.0001. HDL = high-density lipoprotein; LDL = low-density lipoprotein; TG = triglycerides; TC = total cholesterol; BG = blood glucose; PGC-1α = peroxisome proliferator-activated receptor-gamma coactivator (PGC)-1alpha; TNF-α = tumor necrosis factor-alpha; IL-10 = interleukin 10; PRMT-1 = protein arginine N-methyltransferase 1; DDAH-II = dimethylarginine dimethylaminohydrolase 2; ADMA = plasma asymmetric dimethylarginine; CFED: cholesterol- and fat-enriched diet.

Metabolic dysfunction was induced in mice with a cholesterol- and fat-enriched diet (CFED). The AEC extract was administered to assess its effects on various markers associated with cardiometabolic syndrome. The body weight, food intake, water intake, and FER characteristics of the mice are shown in Table 1. The effectiveness of AEC on the cardiometabolic markers is shown in Figure 2.

Figure 2 reveals the capability of AEC in modulating cardiometabolic markers. AEC significantly (*p* < 0.05) improved the blood glucose and lipid profile of mice (for a high dose of AEC, HDL: +23.20%; LDL: −18.64%; TG: −33.00%; TC: −11.11%; BG: −7.760%; Figure 2A). AEC, particularly at a 130 mg/kg BW dose, produced greater improvements in HDL, LDL, and TG in mice that received CFED compared to Groups C and D. However, both doses of AEC had a similar effect on the TC levels. The effect of AEC on BG was similar to that of the normal diet, even though the higher dose of AEC still showed improvements in the BG levels.

The health benefits of AEC are also depicted in Figure 2B. Both AEC treatments were shown to improve cardio SOD serum levels compared to normal and CFED diets. On the other hand, 65 mg/kg BW and 130 mg/kg BW of AEC were proven to reduce amylase and lipase serum levels in mice receiving CFED, with the higher dose of AEC being similar to a normal diet. In Figure 2C, AEC treatments show improvements in the inflammatory markers PGC-1α and IL-10, which significantly increased, although the effect between both doses was similar. While both doses of AEC lowered the TNF-α levels, the higher dose of AEC showed an enhanced effect.

Figure 2D shows that AEC also exhibited positive health implications by improving the expressions of PRMT-I and DDAH-II, in particular, in mice receiving CFED supplemented with 130 mg/kg BW AEC. The higher dose of AEC also lowered ADMA concentration to a level that was similar to a normal diet.

### 3.3. Gut Microbiome Modulation in Mice Administered a Cholesterol- and Fat-Enriched Diet Supplemented with AEC

#### 3.3.1. Effect of AEC on Gut Microbiota Composition

**Figure 3:**
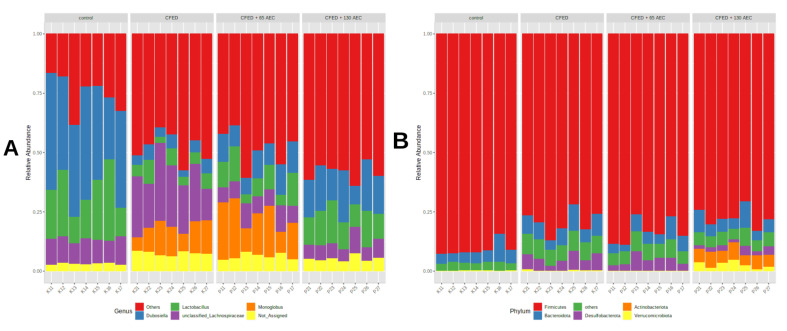
Taxonomic composition at the genus level (**A**) or phylum level (**B**). Each colored bar represents the percentage of each phylum or genus relative to the total microorganisms. CFED: cholesterol- and fat-enriched diet.

After performing low-count and variance filtering, the gut microbiota of the control mice was predominantly composed of *Firmicutes* at the phylum level, which accounted for 91% of the total bacteria. CFED increased the abundance of *Bacteroidota* and *Desulfobacteria* compared to the control mice (Figure 3B). Supplementation with AEC had a different and dose-dependent effect on the gut microbiota community. A low dose of AEC increased the proportion of *Campylobacterota,* while a high dose of AEC increased the proportion of *Actinobacteria* (Figure 3). At the genus level (Figure 3A), control mice were enriched with *Dubosiella* (39%), *Lactobacillus* (20%), and *Lachnospiraceae* (11%), while *Lachnospiraceae* (23%) and *Monoglobus* (11%) were enriched in mice fed a CFED diet. Mice supplemented with low AEC had a high proportion of *Monoglobus* (17%), *Dubosiella* (11%), *Lachnospiraceae* (10%), *Lachnospiraceae* NK4A136 group (9%), and *Lactobacillus* (9%), while the high AEC group had a high proportion of *Dubosiella* (16%), *Lactobacillus* (12%), *Eubacterium fissicatena* group (12%), *Faecalibaculum* (10%), and *Lachnospiraceae* (9%). In particular, the *Bifidobacterium* genus was proportionally higher in the high-dose AEC group compared with other groups.

#### 3.3.2. Effect of AEC on Gut Microbiota Diversity

**Figure 4:**
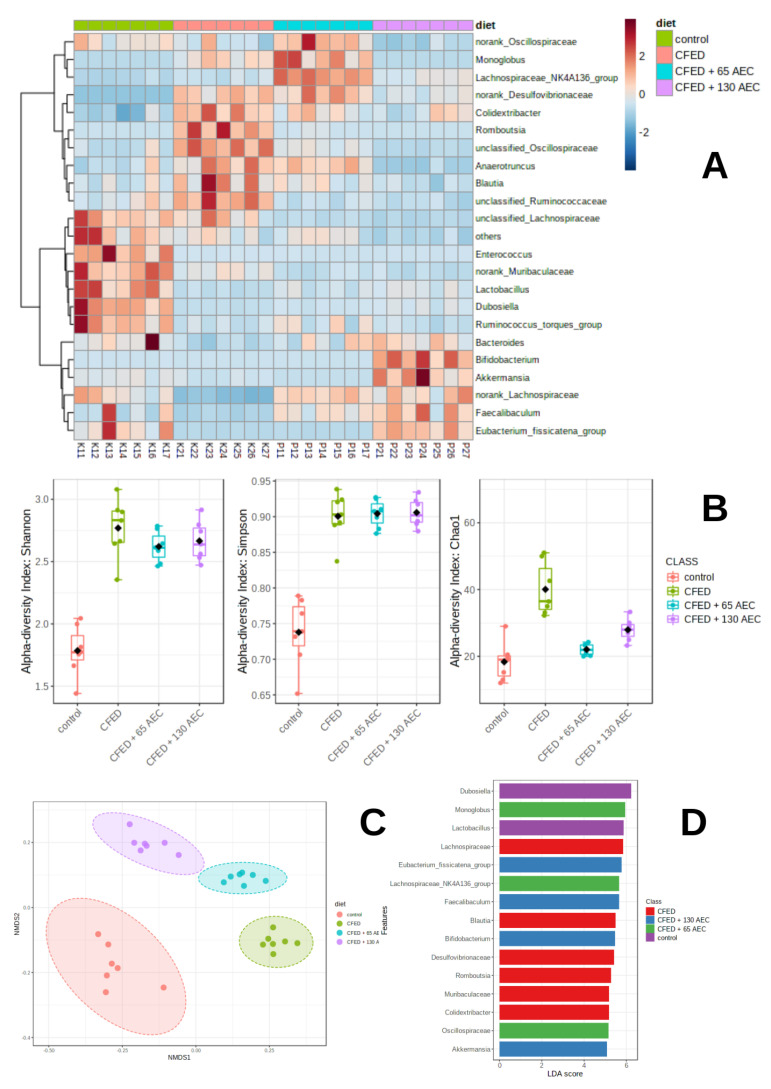
Clustering and community profiling of gut microbiota in mice induced with CFED. Heatmap clustering of individual gut microbiota (bottom) according to diet groups (top) based on Euclidean distance (**A**) Boxplot of distribution of alpha diversity values (Shannon, Simpson, and Chao1) among diet groups (**B**) Non-metric multidimensional scaling (NMDS) plot of all samples using the Bray-Curtis resemblance matrix (**C**) Linear discriminant analysis (LDA) effect size (LEfSe) analyses of gut microbiota according to diet at the genus level; each colored bar indicates a genus that was significantly enriched in each consecutive group (**D**) CFED: cholesterol- and fat-enriched diet.

Heatmap clustering of individual gut microbiota (bottom) according to diet groups (top) was based on Euclidean distance (Figure 4A). A significant difference (*p* < 0.01) was observed in the alpha diversity indices (Shannon, Simpson, and Chao1 indexes) according to the ANOVA test (Figure 4B). Mice fed with CFED had the highest alpha diversity index parameters compared with the other groups. A non-matrix multidimensional matrix (NMDS) using the Bray-Curtis distance was employed to compare the beta diversity among groups (Figure 4C). The results showed that gut microbiota was grouped according to the treatment groups (stress 0.05; *R*^2^ = 0.82). One-way PERMANOVA analysis revealed a significant difference (*p* = 0.001), which indicated distinct gut microbiota communities among the groups.

To identify bacteria that were differentially enriched, linear discriminant analysis (LEfSe) was performed (Figure 4D). The results showed that there were 31 significant features with a log LDA score > 3 and a false discovery rate (FDR) below 0.05. *Dubosiella* and *Lactobacillus* were abundant in the control mice, whereas *Colidextribacter*, *Muribaculaceae*, *Romboutsia*, *Desulfovibrionaceae*, *Blautia,* and *Lachnospiraceae* were enriched in the CFED group. *Monoglobus*, *Lachnospiraceae* NK4A136 group, and *Oscillospiraceae* were enriched in the low-dose AEC group, while the *Eubacterium fissicatena* group, *Faecalibaculum*, and *Bifidobacterium* were enriched in the high-dose AEC group.

**Figure 5:**
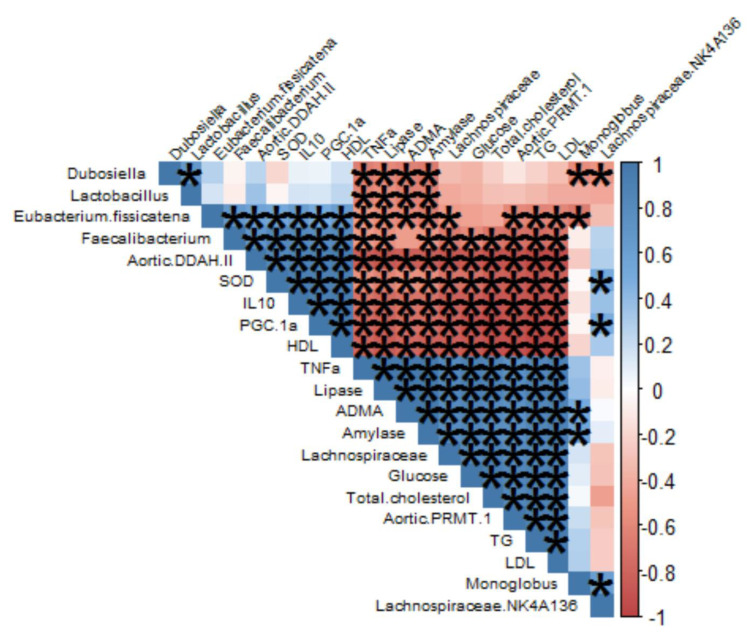
Heatmap of Pearson correlation between the gut microbiome and blood metabolic profiles of mice. The asterisks inside the heatmap indicate *p* < 0.01.

We performed Pearson correlation analysis to investigate the relationship between the abundances of seven significantly altered gut microbial species (FDR < 0.1) and changes in the metabolic biomarker indices in the intervention group (Figure 5). Close associations were found between glucose metabolism, lipid profile, inflammatory cytokines, and the studied microbiome species. *Dubosiella*, *Lactobacillus*, and *Eubacterium fissicatena* showed a negative association with TNF alpha, lipase, ADMA, and amylase. In particular, *Faecalibacterium* was negatively associated with TNF alpha, lipase, amylase, glucose, total cholesterol, aortic-PMRT-1, triglycerides, and LDL-cholesterol. However, *Lachnospiraceae* was positively associated with glucose, total cholesterol, aortic-PMRT-1, and LDL-cholesterol.

## 4. Discussion

*Caulerpa racemosa* is extensively used and has become one of the most popular marine fresh foods because of the various health benefits that it offers. The *Caulerpa* species are known to contain a high amount of macro- and micro-minerals, antioxidants, and secondary metabolites with attractive development or further processing prospects, supported by their distinctive taste and color [21]. Therefore, as an attempt to further advance our understanding of the potential health benefits of this marine alga, we evaluated its activity in vitro, as well as in mice models based on cardiometabolic and microbiome biomarkers. In vitro, *Caulerpa racemosa* showed potent antioxidant activity (DPPH & ABTS radical scavenging activity assays) and inhibition of α-glucosidase, α-amylase, and lipase (Figure 1). These properties are essential for mitigating and alleviating cardiometabolic syndrome. AEC showed EC_50_ values in DPPH and ABTS of 116.9 μg/mL and 121.7 μg/mL, respectively (Figure 1), which were lower than that of glutathione (often called the “master antioxidant” because of its aptitude to exploit the performance of other antioxidants) and Trolox (a water-soluble antioxidant synthesized as a vitamin E derivative with high radical scavenging potential) [22]. This study was also in line with a study by Belkacemi, where *Caulerpa racemosa* extract from Algeria exhibited an antioxidant capacity that was comparable to the controls used here [23]. The high antioxidant capacity may be due to the phytochemicals contained in *Caulerpa racemosa,* especially phenolics and flavonoids. These compounds contain hydroxyl groups, which function as hydrogen donors to stabilize free radicals [24]. Moreover, non-polar compounds such as glycolipids, phospholipids, steroids, terpenes, carotenoids, and fatty acids (especially PUFAs) are also thought to contribute to the overall radical-scavenging capacity of AEC [25]. Free radical-neutralizing capacity is essential for treating cardiometabolic syndrome because the increase in oxidative stress is deeply involved in the pathogenicity of hypertension and atherosclerosis. Pro-inflammatory adipokines caused by obesity also produce an immense amount of oxidative stress, which contributes to the progression of cardiometabolic syndrome [26]. Furthermore, this study also utilized ultrasound-assisted extraction, which is often used to increase extraction efficiency as well as natural ingredient bioactivity. Furthermore, since this technique is regarded as a “green extraction,” it is environmentally friendly, requiring minimal use of solvent and time [27]. α-Amylase, α-glucosidase, and lipase are the main enzymes involved in the hydrolyzation process of carbohydrates and fat. Therefore, reacting these enzymes with AEC would cause lowered absorption of the mentioned dietary molecules, hinder carbohydrate digestion, prevent diet-induced obesity and hyperglycemia, and control diabetes, subsequently lowering the risks of micro-and macrovascular complications [28]. In addition, α-glucosidase inhibition can also aid in gastric emptying, causing satiety and weight loss, which are useful properties in obesity treatment [29]. α-Glucosidase and α-amylase inhibition by AEC was comparable to that by acarbose, an anti-diabetic drug, while lipase inhibition also showed similar results to those of orlistat, a medication used to treat obesity. This shows the potential of AEC as an alternative route of therapy.

To further advance and validate the previously mentioned results, an in vivo study using animal models was performed to assess the effect of *C. racemosa*. Data showed that the CFED diet intervention significantly increased both the final body weight and weight gain per day of mice. However, the food and water intake of the CFED mice was about the same as that of the other groups. Thus, the observed increases may be due to the significantly increased FER of the CFED-only group (Table 1). By contrast, *C. racemosa* groups experienced significantly less weight gain per day and FER compared to the other groups. This weight-loss potential is in line with other studies involving other species of *Caulerpa* (*Caulerpa sertularioides* and *Caulerpa prolifera*), which showed a significant inhibitory effect on adipogenesis of preadipocytes *in vitro*, with reductions of lipogenic transcription factors such as PPARγ, C/EBPβ, and C/EBPα mRNA [30]. Applying a similar study model, *Caulerpa lentillifera* intervention on CFED rats resulted in lower body weight post-treatment (although the results were not significant), as well as a lower FER compared to the other groups [31].

Alterations in the free fatty acid metabolism and adipose tissue dysregulation are the main suspects of hyperglycemia and dyslipidemia in cardiometabolic syndrome. The uncontrolled release of free fatty acids from adipose tissues to the bloodstream can impair the activity of insulin during muscle glucose uptake stimulation. Excess adipose tissue in obesity also causes an adipose tissue hypoxia state, which leads to insulin resistance, inflammation, and adipocyte apoptosis, as well as uncontrolled lipid secretion [32]. Furthermore, the buildup of free fatty acids in the liver (otherwise known as ectopic lipids) increases hepatic VLDL and plasma TG concentration. An increase in plasma TG leads to the transfer of TGs from VLDL to HDL, increasing HDL clearance, which in turn leads to reduced HDL and higher LDL concentrations [33]. Cardiometabolic markers were assessed in this study, and AEC intervention significantly improved the lipid profile and blood glucose in vivo. HDL increase together with LDL and TG decrease was found to be dose-dependent; however, no significant differences were found between doses in the TG and blood glucose parameters. In line with the in vitro results, lipase and amylase serum were significantly reduced in CFED mice that were given AEC, and the reduction was dose-dependent. The improvement in the lipid and blood glucose profiles can be explained by the inhibition of the digestive enzyme, as seen in vitro and in vivo. In the lipid metabolism process, inhibition of lipase by AEC prevents the conversion of ingested lipids in the form of TG into monoglycerides and fatty acids. Therefore, fewer lipid products are diffused across the intestinal cell membranes and reach the blood circulation, thus reducing excess lipid metabolism, fat deposition, and body weight and improving the lipid profile [34]. This offers the potential benefit of alleviating “the triad” of dyslipidemia, as seen in cardiometabolic syndrome, which is the increase in serum TG and LDL particles together with depressed levels of HDL. Improvement of the lipid profile can reduce the risk of atherosclerotic vascular changes, coronary artery disease, and major cardiovascular events [35]. In addition, inhibition of both α-amylase and α-glucosidase by AEC lengthens the duration of carbohydrate digestion and reduces postprandial glucose. This occurs because oligosaccharides entering the gastrointestinal tract depend on α-amylase to be broken down into smaller molecules such as maltose and maltotriose. Meanwhile, α-glucosidase carries out this process by cleaving disaccharides into digestible monosaccharides [34]. Altered glucose metabolism and CVD risk are both deeply related, as multiple studies have shown hyperglycemia to be a key risk factor for cardiovascular and all-cause mortality [36]. The control of blood glucose that is offered by AEC can be beneficial, preventing continuous hyperglycemia that leads to micro (neuropathy, nephropathy, retinopathy) and macrovascular complications (peripheral vascular disease, myocardial infarction, and stroke) [37].

The antioxidant status of mice treated with AEC, determined in vitro, was supported by in vivo findings of a significant SOD cardio serum increase in the AEC groups, although no significant difference was found between the dose groups. SODs are recognized as the “front line” of defense against reactive oxygen species (ROS)-mediated injury. These metalloenzymes catalyze the conversion of the superoxide anion (O_2_^−^) free radical into hydrogen peroxide (H_2_O_2_) and molecular oxygen [38]. In a hyperglycemic state, endothelial cells increase the production of O_2_^−^ while immoderate amounts of O_2_^−^ lead to the inhibition of glyceraldehyde 3-phosphate dehydrogenase (a glycolytic pathway enzyme). This causes glucose accumulation as well as increased shifts to various alternative pathways of glucose metabolism, eventually leading to the production of advanced glycation end products [39]. Moreover, ROS are also responsible for pathological processes in the vasculature, causing endothelial dysfunction through the interruption of vasoprotective pathways such as NO signaling, as well as triggering inflammasome and cytokines such as IL-1β and IL-8 via activation of caspase-1. The mechanisms above trigger atherosclerosis [40]. By increasing SOD in serum, the aforementioned consequences of cardiometabolic syndrome can potentially be prevented.

Inflammatory biomarkers were evaluated in this study. TNF-α was significantly reduced by AEC intervention. Reduction in TNF-α was dose-dependent, and a significant increase in IL-10 and PGC-1α was found. However, no significant differences in IL-10 and PGC-1α were found between the dose groups. PGC-1α is known as the master regulator of mitochondrial processes such as oxidative phosphorylation and ROS detoxification [41]. Furthermore, PGC-1α also regulates mitochondrial antioxidant gene expression, even though its dysregulation at low levels triggers an inflammatory response as well as oxidative stress and promotes the NF-κB pathway, which is responsible for inducing pro-inflammatory genes [42]. Various inflammatory markers have been determined to be deeply associated with significant risks of diabetes and cardiovascular disease. Obesity itself, as one of the main features of cardiometabolic syndrome, promotes the generation of pro-inflammatory factors, including TNF-α, in turn promoting insulin resistance [43]. Moreover, inflammatory cytokines also act on the liver and increase VLDL production, deactivating liver X receptors and causing increased cholesterol accumulation [44].

A possible mechanism implied in the development of endothelial dysfunction and oxidative stress is an increase in the levels of asymmetrical dimethylarginine (ADMA). ADMA circulation levels are increased in endothelial dysfunction-related diseases such as hypertension, hyperlipidemia, and diabetes [45]. ADMA itself is classified as a major endogenously derived methylated arginine residue that acts by inhibiting nitric oxide synthase (NOS). Nitric oxide is a well-known and potent vasodilator that is essential in cardiovascular homeostasis by exerting anti-atherogenic and anti-proliferative activities on the vasculature [46]. Furthermore, ADMA is also known to elevate ROS levels, causing oxidative stress. ADMA is synthesized in the heart, smooth muscle, and endothelial cells by PRMT-1, which is a group of enzymes that performs the methylation process [47]. Meanwhile, most (80–90%) of ADM is metabolized by DDAH, which is relevant to this study. DDAH-2 is highly expressed in vascular muscle cells and the endothelium, especially in tissues containing NOS [48]. This study also evaluated the PRMT-DDAH-ADMA metabolic axis expression in response to the CFED and AEC intervention. PRMT-1 expression and ADMA concentrations were significantly lowered in CFED mice given AEC, while DDAH-2 was significantly increased in the AEC groups, with dose-dependent effects in all parameters. Decreased DDAH activity is closely related to endothelial dysfunction and is suspected to be the mechanism responsible for ADMA-mediated NOS impairment [46]. Therefore, AEC has the potential to ameliorate the mentioned pathological metabolic axis and prevent the development and progression of cardiometabolic syndrome.

Our study also found that AEC supplementation affects gut microbiome diversity. Furthermore, at the taxa levels, the gut microbiota composition also differed significantly. *Dubosiella* and *Lactobacillus* were abundant in the control mice, whereas *Colidextribacter*, *Muribaculaceae*, *Romboutsia*, *Desulfovibrionaceae*, *Blautia*, and *Lachnospiraceae* were enriched in the CFED group. *Lachnospiraceae* comprises 58 genera [49]. All microbiota members of *Lachnospiraceae* are characterized by anaerobic, fermentative, and chemoorganotrophic features, and some display strong hydrolyzing activities (e.g., through the activity of β-xylosidase, α- and β-galactosidase, α- and β-glucosidase, or α-amylase) [50]. Several studies have demonstrated that *Lachnospiraceae* (including *Blautia*) are upregulated during diabetes-associated obesity [51] and during the development of non-alcoholic fatty liver disease (NAFLD) [52]. The increase in *Lachnospiraceae* (including *Blautia*) in these metabolic disorders may be partly explained by hyperglycemia that often occurs during obesity-related diabetes as well as NAFLD [51,53]. Likewise, our correlation analysis indicates that *Lachnospiraceae* was positively associated with fasting glucose levels.

*Monoglobus*, *Lachnospiraceae* NK4A136 group, and *Oscillospiraceae* were enriched in the low-dose AEC group, while the *Eubacteria* group, *Faecalibaculum*, and *Bifidobacterium* were enriched in the high-dose AEC group. These results suggest that the effect of AEC on the gut microbiome might be dose-dependent. A study suggested that *Caulerpa racemosa* exhibits antimicrobial activity that may inhibit pathogenic bacteria [54]. In this study, a high dose of AEC increased the SCFA producers such as *Faecalibacterium* and *Bifidobacterium*. In addition, these increases were negatively associated with the inflammatory marker (TNF-α), ADMA, and lipid profiles and positively associated with an increase in the antioxidant capacity (SOD levels) and PGC-1α levels in the blood. This suggests that the AEC effect on the regulation of inflammation-associated pathways as well as lipid-glucose regulation, may also be mediated by the modulation of *Faecalibacterium* and *Bifidobacterium* [55,56,57]. This shows that the Caulerpa genus has the potential to become a superfood, in line with a recent study showing its potential anti-non-communicable diseases properties, such as obesity-related diseases [58].

## 5. Conclusions

The aqueous extract of *Caulerpa racemosa* (AEC) exhibited potential antioxidant properties in vitro. Lipase inhibition, α-amylase, and α-glucosidase activities were observed. Thus, AEC is a promising functional ingredient with potential anti-cardiometabolic syndrome effects. The observed trends of the PRMT-1/DDAH/ADMA pathway and gut modulation of the microbiota demonstrated positive effects against cardiometabolic syndrome, achieved by dietary supplementation with a high dose (130 mg/kg Body Weight [BW]) of AEC. Human clinical trials are still needed and are being planned for the foreseeable future.

## 6. Patents

The preparation method and formulation of an aqueous extract of *Caulerpa racemosa* (AEC) resulting from the work reported in this study have been registered as a patent in Indonesia with number S00202211473 (Fahrul Nurkolis is a patent holder of the AEC Extract).

## Figures and Tables

**Table 1:** Body weight, feed, water intake, and FER characteristics of experimental mice (*Mus musculus*).

Groups	Normal	CFED	C	D	*p* ^b^
Initial body weight (g)	22.55 ± 1.717	22.05 ± 2.203	22.03 ± 1.033	22.42 ± 2.908	0.9231
Final body weight (g)	65.59 ± 3.606	82.27 ± 4.206	48.66 ± 5.509	43.18 ± 4.481	<0.0001
*p* ^a^	*<0.0001*	*<0.0001*	*<0.0001*	*<0.0001*	
Weight gain (g/day)	0.9357 ± 0.08751	1.309 ± 0.07279	0.5789 ± 0.1116	0.4513 ± 0.1338	<0.0001
Food intake (g)	5.204 ± 0.6179	5.116 ± 0.8741	5.037 ± 1.168	5.009 ± 0.6075	0.9556
Water intake (mL)	5.758 ± 0.6237	5.752 ± 0.8913	5.349 ± 1.001	5.208 ± 0.5297	0.2954
FER (%)	18.18 ± 2.413	26.32 ± 5.064	12.00 ± 3.480	9.011 ± 2.496	<0.0001

^a^ Dependent or paired *t*-test, CI 95% (0.05). ^b^ ANOVA CI 95% (0.05). The letter (a) behind the number in the same row indicates non-significant results. Food efficiency ratio (FER, %) = (body weight gain of experimental mice (g/day)/food intake (g/day)) × 100. CFED: cholesterol- and fat-enriched diet. C: Mice group were given a CFED diet and water ad libitum with daily supplementation of 65 of body-weight (BW) AEC. D: Mice group were given a CFED diet and water ad libitum with daily supplementation of 130 mg/kg of body-weight (BW) AEC.

## Data Availability

The data datasets generated and/or analyzed in this study are available on request from the corresponding author.

## Supplementary Materials

The following supporting information can be downloaded at: https://www.mdpi.com/article/10.3390/nu15040909/s1, Table S1. High-Performance Liquid Chromatography-Mass Spectrometry (HPLC-MS) of Caulerpin in Aqueous Extract of *Caulerpa racemosa* (AEC).
