# Supplementary material for: Dietary Supplementation of Caulerpa racemosa Ameliorates Cardiometabolic Syndrome via Regulation of PRMT-1/DDAH/ADMA Pathway and Gut Microbiome in Mice
Source: Nutrients. 2023 Feb 11;15(4):909. doi: 10.3390/nu15040909 (PMC9959712; doi:10.3390/nu15040909)
Supplement: Supplementary file 1 [file nutrients-15-00909-s001.zip › nutrients-2140323-supplementary.pdf]

Supplementary File

Table S1. High-Performance Liquid Chromatography-Mass Spectrometry (HPLC-MS) of Caulerpin in Aqueous Extract of *Caulerpa racemosa* (AEC).

Compound (PubChem ID)	Molecular Formula	Observed m/z	Category	Chemical structure depiction of caulerpin
Caulerpin (5326018)	$C_{24}H_{18}N_2O_4$	398.13278	an indole alkaloid	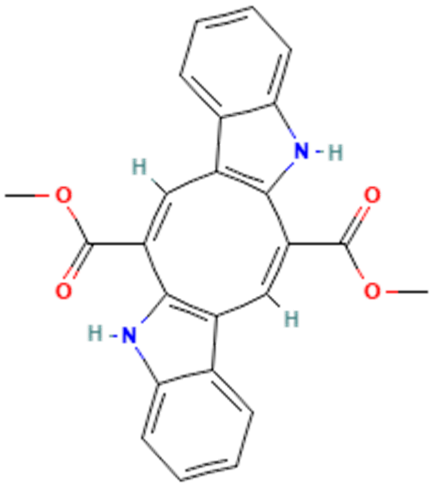<br>National Center for Biotechnology Information. PubChem Compound Summary for CID 5326018, Caulerpin. https://pubchem.ncbi.nlm.nih.gov/compound/Caulerpin. Accessed 30 January 2023

Test on caulerpin in the extract (sample) was carried out using liquid chromatography–mass spectrometry (LC-MS), and a mass of 398.13278 (397.12257 m/z) was obtained. This shows that there is an antioxidant compound caulerpin in the sample extract.
